# Adverse pregnancy outcomes and imbalance in angiogenic growth mediators and oxidative stress biomarkers is associated with advanced maternal age births: A prospective cohort study in Ghana

**DOI:** 10.1371/journal.pone.0200581

**Published:** 2018-07-17

**Authors:** Enoch Odame Anto, William K. B. A. Owiredu, Samuel Asamoah Sakyi, Cornelius Archer Turpin, Richard K. D. Ephraim, Linda Ahenkorah Fondjo, Christian Obirikorang, Eric Adua, Emmanuel Acheampong

**Affiliations:** 1 Department of Molecular Medicine, School of Medical Sciences, Kwame Nkrumah University of Science and Technology (KNUST), Kumasi, Ghana, West Africa; 2 School of Medical and Health Sciences (SMHS), Edith Cowan University (ECU), Perth, Western Australia, Australia; 3 Department of Obstetrics and Gynaecology, Komfo Anokye Teaching Hospital, Kumasi, Ghana, West-Africa; 4 Department of Medical Laboratory Technology, University of Cape Coast, Cape Coast, Ghana, West Africa; University of Missouri Columbia, UNITED STATES

## Abstract

**Background:**

Advanced maternal age (AMA) has been associated with negative pregnancy outcomes. Oxidative stress (OS) and defective placental dysfunction are contributing factors. This study determined the association between AMA and adverse pregnancy outcomes, OS biomarkers and angiogenic growth mediators (AGMs) in normal pregnancies.

**Methods:**

This prospective cohort study conducted at the Obstetrics and Gynaecology (O&G) Department of the Komfo Anokye Teaching Hospital (KATH) finally included 175 normal pregnant women comprising, 58 AMA (35–45 years), 55 (30–34 years) and 62 optimal childbearing age (20–29 years). Venous blood samples were collected at 28–32 weeks for soluble fms-like tyrosine kinase-1 (sFlt-1), placental growth factor (PIGF), 8-epiprostaglandinF2-α (8-epi-PGF2α) and total antioxidant capacity (TAC) assays.

**Results:**

Pregnancies of AMA had a significantly higher levels of sFlt-1, 8-epi-PGF2α and 8-epi-PGF2α: PIGF ratio but a reduced level of PIGF, TAC and PIGF: sFlt-1 ratio compared to 20–29 years (p<0.0001). A significant negative correlation between AMA and PIGF (r = -0.294; p = 0.038); TAC (r = -0.215; p = 0.001) and PIGF: sFlt-1 ratio (r = -0.457; p<0.0001) and a positive correlation with sFlt-1 (r = 0.269; p = 0.017), 8-epiPGF2α (r = 0.277; p = 0.029) and 8-epi-PGF2: PIGF ratio (r = 0.461; p<0.0001) levels were observed. The adjusted odds ratio (aOR), and 95% confidence interval, and p value for the significant independent adverse outcomes associated with AMA were emergency caesarean section [21.7 (5.9–121.3), p<00001], elective caesarean section [2.7(0.9–5.8), p = 0.0105], stillbirth [12.6(1.4–82.1), p<0.0001], post-partum haemorrhage [4.3(1.1–18.5), p = 0.0094], preterm delivery [8.2(3.5–28.4), p<0.0001], low birth weight babies [9.7(2.8–29.3), p<0.0001], birth asphyxia [3.8(1.6–12.7), p = 0.0054], Apgar score ≤ 7 after 5 min for babies [10.1(4.7–23.2), p<0.0001], placental abruption [3.5(1.3–8.4), p = 0.0117] and intrauterine growth restriction (IUGR) [4.6(2.3–12.9), p = 0.0001].

**Conclusion:**

AMA pregnancies correlate with adverse pregnancy outcomes and imbalance in OS biomarkers and AGMs. It is incumbent on health care givers to provide effective antenatal care among AMA mothers as early identification of these imbalance and treatment can prevent adverse pregnancy outcomes.

## Introduction

Advanced maternal age (AMA) is defined as pregnancy among women aged 35 years or older. AMA is common worldwide particularly in the United States and South Africa where the prevalence rates per 1000 women are 47.3% and 17.5% respectively [1]. The main reasons for delayed child bearing among many women include: waiting to establish a career or attain higher education before getting married, poverty and remarriage [2, 3]. Unfortunately, studies have shown that AMA could have serious repercussions and it has been linked with many poor pregnancy outcomes including stillbirth [4], preterm birth [5], low birth weight [6] and cesarean delivery [7]. In Ghana, AMA pregnancies were associated with higher rate of stillbirth (13.7%) with less instrumentation delivery [8].

It has long been suggested that ageing is associated with increased inflammation and reactive oxygen species (ROS) which leads to imbalance in both the generation and removal of free radical species (superoxide, hydroxyl radical) and subsequently result in oxidative stress (OS) [9]. However, the exact mechanism(s) linking AMA and pregnancy outcomes are not fully understood. Optimal OS is important in regulating placental angiogenesis for a successful placental vasculature as well as adequate exchange of nutrients and oxygen between mother and foetus [10]. However, increase OS may switch placenta angiogenesis which can result in placental insufficiency and poor pregnancy outcomes [11]. Since placental angiogenesis is regulated by angiogenic growth mediators (AGMs), increase OS is likely to cause an imbalance in these mediators. [12]. A recent study showed that adverse pregnancy outcomes such as intrauterine growth restriction (IUGR), placenta praevia, abruptio placenta, stillbirth and post-partum haemorrhage (PPH) were associated with an increased serum levels of soluble fms-like tyrosine kinas (sFlt-1), 8-epiprostaglandinF2-alpha (8epi-PGF2α) and a corresponding decreased levels of placental growth factor (PlGF) and total antioxidant capacity (TAC) preeclamptic births [12].

However, there is dearth of data to justify whether these imbalances are only linked to hypertensive disorders of pregnancy or are partly associated with AMA pregnancies.

Although ageing is associated with increased OS and that increased OS influences placental angiogenesis, few studies have examined these factors in parallel among AMA pregnancies. Therefore, this present study seeks to determine the association between AMA pregnancy and adverse pregnancy outcomes, OS biomarkers and AGMs among a cohort of normal pregnant women in Ghana.

## Materials and methods

### Study design and study setting

This hospital based prospective cohort study was carried out from April, 2014 to May, 2015 at the Obstetrics and Gynaecology (O & G) Department of Komfo Anokye Teaching Hospital (KATH) in Kumasi. Kumasi has an average population of 4,780,380 (Ghana Statistical service, 2012) and is the capital of the Ashanti Region of Ghana. KATH has over 1000 bed capacity and serves as a major referral center for the middle belt and northern part of Ghana. The total number of antenatal visit is approximately 300 pregnant women per day.

### Recruitment of study participants

A total of 238 normal pregnant women who have registered to attend antenatal clinic at the O&G Department of KATH were recruited and followed after written informed consent was obtained from each participant. Both nulliparous and primiparous pregnant women aged 20–45 years, within the gestational age of ≥ 20–26 weeks with singleton pregnancy were included and followed until birth. During their periodic visits, 63 of the participants did not either meet the inclusion criteria, were lost to follow-up and or did not give consent to participate. For instance, all participants who developed preeclampsia, pregnancy-induced hypertension, heart disease, diabetes mellitus and renal disease during the follow-up period were excluded. A total of 175 participants comprising 58 AMA (35–45 years), 55 (30–34 years) and 62 optimal childbearing age (20–29 years) finally met the inclusion criteria and consented to participate in the study. Blood and urine samples were collected at 28–32 weeks gestation and all pregnancy outcomes were recorded for each participant after birth. Information regarding socio-demographics, clinical and obstetric characteristics were retrieved from maternity ward database. Other additional participant’s information regarding smoking history, contraceptive use, anti-hypertensive drug use, antioxidant supplementations were obtained based on structured closed-ended questionnaire. Physical examination and clinical assessment of participants were performed by a consultant/specialist Obstetrician/Gynaecologist.

### Clinical measurements

A qualified midwife used a mercury sphygmomanometer (Accoson, England) and a stethoscope (3M™ Littmann ® Stethoscopes, USA) to measure the blood pressure of all participants in accordance with the recommendations of the National High Blood Pressure Education Program Working Group, (2000). The procedure was repeated for each patient at 10 minutes intervals. Mean values of duplicate measurements of blood pressure were then recorded and rounded to the nearest 1.0 mmHg. Measurements for blood pressure was done on each antenatal visit.

Height to the nearest centimeter (cm) and weight to the nearest 0.1 kilogram (kg) in light clothing were measured with a bathroom scale (EatSmart ESBS– 07 Precision Series Tracker Digital Bathroom Scale, USA) and a wall-mounted ruler respectively. Body mass index (BMI) was calculated as weight (kg)/ height^2^ (m^2^). BMI was measured for all participants at baseline.

### Blood sample collection and biochemical assays

Ten (10) milliliters (ml) of venous blood sample was collected from each participant by a qualified phlebotomist. Blood was dispensed into serum gel separator tubes (BD vacutainer) and centrifuged (Nüve NF 200, Germany) at 4000 rpm for 30 min. Serum was aliquoted under sterile conditions and stored at −80°C until assay (Thermo Scientific™ Revco™ UxF −Ultra-Low Temperature Freezers, USA).

Serum levels of sFlt-1, PlGF and 8-epi-PGF2α were measured in duplicates using commercially available enzyme linked immunosorbent assay (ELISA) kits from R&D System Inc. (Minneapolis, MN USA). The optical density was measured at 450 nm wavelength using microplate ELISA reader (Mindray MR-96A). The plasma levels of each factor were calculated using standard curves derived from a known concentration of the respective recombinant factors.

TAC reagents were purchased from Green Stone Swiss Co., Ltd, China and levels were estimated spectrophotometrically (Mindray BA-88A, China) at 593nm. The estimation of the ferric reducing ability of plasma (FRAP) was performed using standard protocol as described by Benzie and Strain, (1999). All samples were analysed in triplicates.

### Definition of obstetric terms

Intrauterine growth restriction (IUGR) is defined as poor growth of a fetus whilst in the mother's womb and, it is indicated by an estimated weight below the 10th percentile for its gestational age [13]. Preterm delivery is defined as delivery before 37 completed weeks of gestation [14]. Stillbirth is defined as death prior to the complete expulsion or extraction from its mother of a product of conception, regardless of the duration of pregnancy [14]. An Abruptio placenta is defined as premature separation of normally placed placenta from uterine wall after 20 weeks of gestation and prior to birth [15]. Placenta previa is defined as implantation of the placenta over or near the internal orifices of the cervix after 20 weeks gestation prior to transvaginal or abdominal ultrasonography [16]. Postpartum haemorrhage (PPH) is defined as the loss of more than 500 ml or 1,000 ml of blood within the first 24 hours after childbirth [17]. Birth asphyxia is defined as deprivation of baby’s organs or brain from oxygen before, during or just after birth [18]

### Statistical analyses

Data was entered into Microsoft Excel spreadsheet and statistical analysis was performed using GraphPad Prism software version 5.0 (Graph Pad Software Inc., Los Angeles) for Windows and statistical package for social sciences (SPSS) version 24. The normality of the data was checked using the Kolmogov-Smirnov test. Continuous variables did not assume a Gaussian distribution; hence a non-parametric test was used. Comparison between categorical variables was performed using Chi-square test for two by three or more contingency and Fisher’s exact test for two by two contingency. Kruskal-Wallis test followed by Dunn’s post-hoc multiple comparison test were performed to compare levels of biomarkers in 35–45 years and 30–34 years aged groups to 20–29 years’ groups since the levels of OS and AGMs were not normally distributed. The above statistical analyses were done using GraphPad Prism software version 5.0. Multivariate logistic regression model was performed to identify factors associated with AMA and odds ratios (ORs) plus 95% confidence intervals (CIs) were recorded. Partial correlation between AMA and levels of OS and AGMs was performed after adjusting for confounding factors. Analysis for multivariate logistic regression model and partial correlation was performed using SPSS version 24. Data were reported as median (interquartile range) for continuous variables and as a frequency (percentage) for categorical variables. Statistical significance was accepted at *p< 0*.*05* for all comparisons.

### Ethical consideration

Ethical approval for this study was granted by the Committee on Human Research, Publications and Ethics (CHRPE) (CHRPE/AP/365/14), School of Medical Sciences, Kwame Nkrumah University of Science & Technology (KNUST) and the Research and Development (R&D) Unit of KATH. Written informed consents were obtained from participants before their inclusion into this study. However, participation was voluntary and participants were given the free will to opt out anytime they wanted. Confidentiality of all participants were assured.

## Results

Table 1 shows sociodemographic, obstetric and clinical characteristics of study participants. Maternal age of mothers 30-34years and 35-45years was significantly higher compared to those of 20-29years (p<0.0001). Higher proportion of pregnant women 35-45years were married (p = 0.0013), had completed basic education (p = 0.0043), were Akan’s by ethnicity (p = 0.0374), and were employed (p = 0.5307) compared to 20–29 years. A high proportion of pregnant women 20-29years were nulliparous while AMA mothers were mostly primiparous (p<0.0001). A higher proportion of pregnant women 20–29 years were primigravida while most of the AMA mothers were secundigravida (p = 0.0011). Majority of pregnant women 20–29 years earned low income while higher proportion of those in both 30–34 years and 35-45years earned middle income (p = 0.0265). Pregnant women 35–45 years had significantly higher BMI (p = 0.0390) and delivered preterm (p = 0.0003) compared to those in 20–29 years. There were no statistically significant differences in proportion between systolic blood pressure (p = 0.827), diastolic blood pressure (p = 0.531), gestation age at baseline (p = 0.527), as well as gestation age at blood sampling (p = 0.881) among the maternal age groups.

**Table 1:** Sociodemographic, obstetric and clinical characteristics of study participants.

Variables	20–29 year	30–34 year	35–45 year	p-value
	(n = 62)	(n = 55)	(n = 58)	
**Mean Age (years)**	27.6 (20–29)	31.5 (30–34)*	37.3 (35–45)***	<0.0001^k^
**Marital Status**				0.0013^c^
Married	44(71.0%)	48(87.3%)	55(94.8%)	
Never married	18(29.0%)	7(12.7%)	3(5.2%)	
**Level of education**				0.0043^c^
Never schooled	3(4.8%)	2(3.6%)	4(6.9%)	
Basic	19(30.6%)	23(41.8%)	37(63.8%)	
Secondary	31(50.0%)	18(32.7%)	12(20.7%)	
Tertiary	9(14.5%)	12(21.8%)	5(8.6%)	
**Ethnicity**				0.0374^c^
Akan	38(61.2%)	35(36.6%)	42(72.4%)	
Ga-Adangbe	8(12.9%)	13(23.6%)	5(8.6%)	
Ewe	2(3.2%)	3(5.5)	4(6.9%)	
Northerner	14(22.6%)	4(7.3%)	7(12.1%)	
**Socioeconomic income**				0.0265 ^c^
Low	27(61.2%)	12(36.6%)	14(72.4%)	
Middle	21(12.9%)	33(36.6%)	28(72.4%)	
High	14(3.2%)	10(5.5)	16(6.9%)	
**Occupation**				0.5307^c^
Unemployed	17(27.4%)	14(25.5%)	11(19.0%)	
Employed	45((72.6%)	41(74.5%)	47(81.0%)	
**Parity**				<0.0001^c^
Nulliparous	38(61.3%)	15((27.3%)	13(22.4%)	
Primiparous	24(38.7%)	40(72.7%)	45(77.6%)	
**Gravidity**				0.0011^c^
Primigravida	43(69.4%)	32(58.2%)	21(36.2%)	
Secundigravida	19(30.6%)	23((41.8%)	37(63.8%)	
**SBP (mmHg)**	116.3 (109–118.4)	115(103–118)	114.8(105–118.3)	0.827^k^
**DBP (mmHg)**	71.9(67.3–81.5)	69.8 (66.3–78.6)	70.8 (68.2–77.4)	0.531^k^
**BMI (Kg/m2)**	22.8 (18.3–24.7)	23.9 (19.2–24.5)	24.5 (18.7–25.1)*	0.039^k^
**GA at baseline (wks.)**	22.6 (20.5–25.8)	23.0 (20–25.4)	22.8 (20.7–25.2)	0.527^k^
**GA at BS (wks.)**	29.9 (28–30.7)	30.3 (28.1–31.0)	29.5 (28.3–30.9)	0.881^k^
**GA at delivery (wks.)**	38.3 (36–39.9)	36.7 (32–37)	34.3 (33–35)***	0.0003^k^

Values are presented as frequency (percentage); median (interquartile range); SBP: systolic blood pressure; DBP: diastolic blood pressure; GA: gestational age; BS: blood sampling.

^k^Kruskal-Wallis followed by Dunn’s multiple comparison

^c^ p-value for Chi-square test.

Significant *p<0.05; moderately significant **p<0.001; highly significant ***p<0.0001.

Table 2 shows the serum levels of AGMs and OS biomarkers in relation to maternal age. Pregnant women of AMA (35-45years) had a significantly higher levels of sFlt-1 (p = 0.0001), 8-epi-PGF2α (p<0.0001), and 8-epi-PGF2α: PIGF ratio (p = 0.0003) compared to those of optimal childbearing age (20–29 years). Meanwhile, levels of PIGF (p<0.0001), TAC (p = 0.0017) and PIGF: sFlt-1 ratio (p<0.0001) were significantly lower among pregnant women of 30–34 years and 35–45 years compared to those in 20–29 years. Levels of TAC were significantly lower among pregnant women of 35–45 years compared to those in 20–29 years (p = 0.0017).

**Table 2:** Serum levels of AGMs and OS biomarkers in relation to maternal age.

Study Groups	Maternal age groups (years)			*p-value*
	(20–29)	(30–34)	(35–45)	
PIGF (pg/ml)	157.6 (114.5–172.5)	148.7 (79.3–177.3)	115.1 (88.88–194.7)***	<0.0001
sFlt-1 (pg/ml)	59.0 (37.4–110.6)	87.3 (38.5–174.0)	139.3 (77.10–195.4)***	0.0001
8-epi-PGF2α (pg/ml)	28.1 (23.1–38.9)	38.4 (28.7–60.4)	43.4 (29.58–69.45) ***	<0.0001
TAC (mmol/l)	1.9(0.9–1.25)	1.1 (0.9–1.1)	0.7 (0.55–0.83)**	0.0017
PIGF: sFlt-1 ratio	3.8 (1.0–6.7)	2.0 (0.57–2.9)	0.8 (0.35–1.46)***	<0.0001
8-epi-PGF2α: PIGF ratio	0.2(0.05–0.45)	0.3(0.03–0.61)	0.4(0.07–0.83)***	0.0003

Values are presented as Median (interquartile range). PIGF: Placental growth factor; sFlt-1: soluble fms-like tyrosine kinase; 8-epi-PGF2α; 8-epiprostaglandinF2alpha; TAC: total antioxidant capacity

Kruskal Wallis Test followed by Dunn’s multiple comparisons to compare 30-34years and 35-45years to 20-29years age group.

Significant *p<0.05; moderately significant **p<0.001; highly significant ***p<0.0001.

Table 3 shows partial correlation between maternal age and OS biomarkers and AGMs. After adjusting for maternal age, gravidity, parity, socioeconomic income and BMI, there was a significant negative correlation between mothers 35–45 years of age and levels of PIGF (r = -0.294; p = 0.038); TAC (r = -0.215; p = 0.001) and PIGF: sFlt-1 ratio (r = -0.457; p<0.0001). Meanwhile, a significant positive correlation was observed between mothers’ age 35–45 years and serum levels of sFlt-1 (r = 0.269; p = 0.017), 8-epiPGF2α (r = 0.277; p = 0.029) and 8-epiPGF2: PIGF ratio (r = 0.461; p<0.0001). Additionally, there was a significant negative correlation between mothers’ age 30–34 years and PIGF: sFlt-1 ratio (r = -0.227; p = 0.048)

**Table 3:** Spearman rho partial correlation between maternal age and OS biomarkers and AGMs.

Variables	Maternal age groups (years)		
	20–29	30–34	35–45
**PIGF (pg/ml)**			
r	-0.125	-0.178	-0.294
p-value	0.203	0.086	**0.038**
**sFlt-1 (pg/ml)**			
r	0.062	0.042	0.269
p-value	0.528	0.713	**0.017**
**8-epiPGF2α (pg/ml)**			
r	0.101	0.110	0.277
p-value	0.132	0.293	**0.029**
**TAO-C (mmol/l)**			
r	-0.082	-0.186	-0.215
p-value	0.771	0.107	**0.001**
**PIGF: sFlt-1 ratio**			
r	-0.127	-0.227	-0.457
p-value	0.395	**0.048**	**<0.0001**
**8-epiPGF2: PIGF ratio**			
r	0.104	0.107	0.461
p-value	0.097	0.316	**<0.0001**

r = correlation coefficient. Maternal age, gravidity, parity, socioeconomic income and BMI adjusted partial correlation

Table 4 shows and association between adverse pregnancy outcomes and maternal age.

**Table 4:** Association between adverse pregnancy outcome and maternal age.

	20–29 years	30–34 years	35–45 years		AMA			
Variables	(n = 62)	(n = 55)	(n = 58)	p-value^F^	cOR (95% CI)	p-value	aOR (95% CI)	p-value
**Mode of delivery**								
SVD	53 (85.5%)	39(70.9%)	8(13.8%) ***	<0.0001	reference		reference	
Emergency CS	2(3.2)	7(12.7%)	33(56.9%) ***	<0.0001	39.6(8.8–177.8)	<0.0001	21.7(5.9–121.3)	<0.0001
Elective CS	7(11.3%)	5(9.1%)	17(29.3%) **	0.0058	3.3(1.2–8.6)	0.0213	2.7(0.9–5.8)	0.0105
**Stillbirth (Yes)**	1(1.6%)	5(10.0%)	12(20.7%) **	0.0020	15.9(1.9–126.7)	0.0008	12.6(1.4–82.1)	<0.0001
No					reference		reference	
**Prolonged labour (Yes)**	5(8.1%)	12(21.8%)	8(13.8%)	0.1044	1.8(0.6–5.9)	0.3848	1.1(0.3–3.2)	0.7150
No					reference		reference	
**PPH (Yes)**	3(4.8%)	8(14.5%)	15(25.9%) **	0.0053	6.9(1.9–25.2)	0.0016	4.3(1.1–18.5)	0.0094
No					reference		reference	
**Preterm delivery (Yes)**	15(24.2%)	21(38.2%)	45(77.6%) ***	<0.0001	10.9(4.7–25.3)	<0.0001	8.2(3.5–28.4)	<0.0001
No					reference		reference	
**Low birth weight (Yes)**	5(8.1%)	8(14.5%)	26(44.8%) ***	<0.0001	9.3(3.2–26.5)	<0.0001	9.7(2.8–29.3)	<0.0001
No					reference		reference	
**Birth asphyxia (Yes)**	8(12.9%)	11(20.0%)	28(48.3%) **	0.0001	6.3(2.6–15.6)	<0.0001	3.8(1.6–12.7)	0.0054
No					reference		reference	
**APGAR score ≤ 7 after 5 min (Yes)**	11(17.7%)	21(38.2%)*	44(75.8%) ***	<0.0001	14.6(6.0–35.4)	<0.0001	10.1(4.7–23.2)	<0.0001
No					reference		reference	
**Placental praevia (Yes)**	7(11.3%)	9(16.4%)	15(25.9%)	0.1072	2.7(1.1–7.4)	0.0578	1.5(1.3–7.5)	0.1083
No					reference		reference	
**Placental abruption (Yes)**	6(9.7%)	13(23.6%)	18(31.0%) *	0.0143	4.2(1.5–11.5)	0.0054	3.5(1.3–8.4)	0.0117
No					reference		reference	
**IUGR (Yes)**	4(6.5%)	6(10.9%)	16(27.6%) **	0.0088	5.5(1.7–17.7)	0.0027	4.6(2.3–12.9)	0.0001
No					reference		reference	

Values are presented as frequency (percentage); crude odds ratio (cOR): crudes odds ratio; adjusted odds ratio (aOR).95% CI: 95% confidence interval. IUGR: intrauterine growth retardation; PPH: postpartum haemorrhage; SVD: spontaneous vagina delivery; CS: caesarean section; APGAR: appearance, pulse; grimace, activity and respiration. AMA: advanced maternal age. P-value ^F^: p-value for Fischer’s exact test (comparison between 20–29 years and 35-45years group). Multivariate logistic regression models adjusted for maternal age, parity, gravidity, socioeconomic income and BMI. Significant *p<0.05; moderately significant **p<0.001; highly significant ***p<0.0001.

A significantly higher proportions of pregnant women 35–45 years were associated with both elective (p = 0.0058) and emergency caesarean section (p<0.0001), stillbirth (p = 0.0020), post-partum haemorrhage (p = 0.0053), delivered preterm (p<0.0001), low birth weight babies (p<0.0001), birth asphyxia (p = 0.0001), APGAR score ≤ 7 after 5 min for babies (p<0.0001), Placental abruption (p = 0.0143) and IUGR (p = 0.0088) compared to those in 20–29 year. After adjusting for maternal age, parity, gravidity, socioeconomic income and BMI multivariate logistic regression, it was shown that emergency caesarean section [aOR (95%CI) = 21.7(5.9–121.3), p<0.0001], elective caesarean section [aOR (95%CI) = 2.7(0.9–5.8), p = 0.0105], stillbirth [aOR (95%CI) = 12.6(1.4–82.1), p<0.0001], post-partum haemorrhage [aOR (95%CI) = 4.3(1.1–18.5), p = 0.0094], preterm delivery [aOR (95%CI) = 8.2(3.5–28.4), p<0.0001], low birth weight babies [aOR (95%CI) = 9.7(2.8–29.3), p<0.0001], birth asphyxia [aOR (95%CI) = 3.8(1.6–12.7), p = 0.0054], APGAR score ≤ 7 after 5 min for babies [aOR (95%CI) = 10.1(4.7–23.2), p<0.0001], placental abruption [aOR (95%CI) = 3.5(1.3–8.4), p = 0.0117] and IUGR [aOR (95%CI) = 4.6(2.3–12.9), p = 0.0001] were significant independent risk factors associated with pregnancies of AMA.

## Discussion

This study demonstrated a significant association between AMA and adverse pregnancy outcomes as well as imbalances in AGMs and OS biomarkers.

Particularly for OS biomarkers, there were alterations in levels as depicted by a significantly increased 8-epi-PGF2α and reduced TAC levels in AMA mothers compared to those of optimal childbearing age (20–29 years). Ageing is associated with increased inflammatory response and ROS production; hence increased 8-epi-PGF2α and reduced TAC in AMA mothers are indicative of lipid peroxidation and a compromised antioxidant system respectively [9]. Although pregnancy is physiologically associated with ROS production, pregnant women of AMA are more likely to suffer an increased ROS and OS as increased lipid peroxidation alone could not induce increased antioxidant mechanisms. This finding is typical of a hypoxic placental origin as placental hypoxia is known to induce tissue OS, apoptosis, necrosis, inflammatory response and endothelial dysfunction, all of which affect OS balance [11]. Despite the reduced antioxidant system observed among AMA mothers in this study, our participants were not on antioxidant nutrient supplements, therefore this may have increased an oxidant attack. This study hypothesized that AMA pregnancies are associated with increased OS, which is more likely to affect maternal and foetal wellbeing. Hence, antioxidant supplementation especially during the second and third trimesters will be essential.

We also observed a switch in the balance between proangiogenic (PIGF) and anti-angiogenic growth mediators (sFlt-1) in pregnant women of AMA compared to those with optimal childbearing age. Serum concentrations of PlGF and PlGF/sFlt-1 ratio were significantly reduced with a corresponding increased sFlt-1 levels in pregnant women of AMA compared to those with optimal childbearing age (Table 2). Since PlGF is of placental origin and also play important role in placental angiogenesis, a reduced PlGF and an increased sFlt-1 levels in AMA mothers may depict a defective placental angiogenesis [19, 20]. The mechanism(s) that underpins this finding is not well understood. However, optimal ROS production is known to play a key role in placental angiogenesis signaling pathway and thus the imbalances in PIGF and sFlt-1 observed in AMA pregnancies may have originated from an increased OS [10, 11]. It is possible that the positive association of 8-epi-PGF2α levels with sFlt-1 and inverse association of PIGF and TAC in preeclamptic births reported in our previous study [12] also underlie AMA pregnancies. Since ageing is associated with increased ROS production, which is critical for placental development, the observed imbalance in AGMs could be OS-mediated [9]. However, this link needs further investigation.

Another interesting finding was the significantly increased levels of 8-epi-PGF2α: PIGF ratio in AMA compared to optimal childbearing age pregnancies (Table 2). This finding is novel in the sense that increased OS depicted by increased 8-epi-PGF2α levels may have compromised the PIGF function. Increased OS in this study is due to hypoxic placenta originating from placental insufficiency and incomplete maternal artery remodeling, both of which, are known to induce alterations in placental angiogenesis [10]. Hence OS and AGMs play synergistic roles in reproductive ageing health.

The dynamism of circulatory biochemical markers of OS and AGMs and their relation with ageing are not well-understood. This study has demonstrated a significant positive correlation between pregnancies of AMA and serum levels of sFlt-1, 8-epiPGF2α and 8-epiPGF2: PIGF ratio and a significant negative correlation with serum levels of PIGF; TAC and PIGF: sFlt-1 ratio (Table 3). The strength of this association is observed after adjusting for maternal age, gravidity, parity, socioeconomic income and BMI. Our findings hypothesize that pregnancies of AMA are linked with an increased circulatory anti-angiogenic mediators and lipid peroxidation and a reduced pro-angiogenic and antioxidant system. These findings confirm our earlier result that explained the association between AMA pregnancies and imbalances in OS and AGMs. Laopaiboon et al., [21] have indicated that myometrial function deteriorates with age and this could probably explain the link between advanced maternal age-related angiogenic and oxidative stress imbalance.

Adverse pregnancy outcomes such as IUGR, placental abruptio/praevia, stillbirth and postpartum haemorrhage have all been associated with preeclamptic births [12, 22, 23]. However, the mechanisms that underpin adverse pregnancy outcomes with AMA in normal births remains unclear. Per the observations of this study, a higher proportion of adverse pregnancy outcomes were associated with AMA compared to mothers of optimal childbearing age (Table 4). These findings buttress the previous result that AMA is an independent risk factor for pregnancy complications [21, 24]. Particularly, adverse outcomes such as stillbirth, low birth weight (LBW) babies, low Apgar score and IUGR were 15.9, 9.3, 14.6 and 5.5 times increased odds for AMA pregnancies respectively. The findings are consistent with several studies [21, 25, 26]. Changes in OS products such as lipid peroxidation are associated with abnormal placental function and adverse pregnancy outcomes [27]. Thus, the reduced levels of PlGF and TAO-C and increased sFlt-1 and 8-epiPGF2α observed among AMA women in this study are reflections of an increased OS and abnormal placental angiogenesis culminating into stillbirth, low birth weight babies, low APGAR score and IUGR [12, 28]. In addition, increased proportion of low birth weight babies observed among AMA births could be attributed to the increased proportions of IUGR and preterm delivery [1, 29]. Furthermore, post-partum haemorrhage, preterm delivery, neonatal asphyxia, and placental abruption were significant independent factors for AMA pregnancies (Table 4). Although, the mechanisms that underlie these findings are not fully understood, an overall placental underperfusion and impaired artery remodeling have been implicated [30]. Our previous study indicated that imbalance in OS biomarkers and angiogenic regulatory factors were significantly associated with adverse pregnancy outcome in preeclamptic births [12]; hence it is probable that a similar mechanism codes for AMA births as these imbalances were also observed in this present study. Again, our findings that higher proportions of caesarean delivery were associated with AMA births agree with the findings from previous studies [25, 26, 31]. Neonatal asphyxia is associated with impaired placental hypoxia/ischaemia which leads to a reduced oxygen supply [18]. Hence, the higher proportion of still births observed among AMA pregnancies in this study may be due to the higher proportions of neonatal asphyxia.

While the findings in this study are interesting, it cannot be concluded without stating some limitations that may have affected our results. Firstly, the sample size was small and because this was a hospital-based study, our results cannot be generalized for the entire population. Secondly, we could not collect multiple blood samples for biochemical analysis and therefore the pattern of changes in levels of AGMs and OS biomarkers were not observed. Thirdly, due to the lack of age-matched non-pregnant women comparison group, we could not ascertain whether a pre-pregnancy OS levels was responsible for the adverse pregnancy outcome or it was attributed to only pregnancy OS levels. Nonetheless, this study is novel and the first to be conducted in a Ghanaian population. This is a baseline study, hence, future studies on longitudinal cohorts would help to gain additional insights into the molecular mechanisms underpinnings of adverse pregnancy outcomes.

## Conclusion

Adverse pregnancy outcomes and imbalances in angiogenic growth mediators and oxidative stress biomarkers are associated with AMA births. These associations remained significant even after adjusting for possible confounders such as maternal age, parity, gravidity, socioeconomic income and BMI. Pregnancies of AMA may be producing more prooxidants and anti-angiogenic molecules and may be using up antioxidant proteins for its metabolic processes; hence the reduced concentration of TAC and PlGF in circulation. Additionally, the increase in sFlt-1 and 8-epiPGF2α in AMA is an indication that these markers may play a synergistic role in endothelial and placental dysfunction culminating in these adverse pregnancy outcomes. This study is timely and would allow early detection of abnormal biochemical profile to aid early antenatal care and prevent risk for adverse pregnancy outcomes.
